# Identification and phylogenetic analysis of Jingmen tick virus in Jiangxi Province, China

**DOI:** 10.3389/fvets.2024.1375852

**Published:** 2024-05-02

**Authors:** Zirui Liu, Ruiming Hu, Huabin Cao, Peng Huang, Hui Yan, Puyan Meng, Zhiwei Xiong, Xueyan Dai, Fan Yang, Li Wang, Qian Qiu, Linjie Yan, Tao Zhang

**Affiliations:** ^1^Jiangxi Provincial Key Laboratory for Animal Health, Institute of Animal Population Health, College of Animal Science and Technology, Jiangxi Agricultural University, Nanchang, China; ^2^Jiangxi Engineering Research Center for Animal Health Products, College of Animal Science and Technology, Jiangxi Agricultural University, Nanchang, China; ^3^Jiangxi Wildlife and Plant Conservation Center, Nanchang, China; ^4^Jiangxi Academy of Forestry, Nanchang, China; ^5^Jiangxi Biotechnology Vocational College, Nanchang, China

**Keywords:** Jingmen tick virus, ticks, wild boars, Jiangxi Province, phylogenetic analysis

## Abstract

Jingmen tick virus (JMTV) is a newly identified segmented flavivirus that has been recognized in multiple hosts, such as humans, buffalos, bats, rodents, mosquitos and ticks. Various clinical cases and studies manifested that JMTV is a true arbovirus with wide host spectrum and showed potential threats toward public health. JMTV has been reported in multiple countries in Asia, Europe, Africa, and America. Moreover, wild boars serve as an important intermediary between humans and the wild ecological system. In China, it has been reported in nine provinces, while the prevalence and the distribution of JMTV in most regions including Jiangxi Province are still unknown. Thus, to profile the distribution of JMTV in Jiangxi Province, an epidemiological investigation was carried out from 2020 to 2022. In current study, 66 ticks were collected from 17 wild boars in Jiangxi Province. The results showed that 12 out of 66 ticks were JMTV positive, indicating JMTV is prevalent in ticks and boars in Jiangxi Province. The genome sequences of JMTV strain WY01 were sequenced to profile viral evolution of JMTV in China. Phylogenetic analysis divided JMTV strains into two genotypes, Group I and Group II. WY01 belongs to Group II and it shares the closest evolutionary relationship with the Japan strains rather than the strains from neighboring provinces in China suggesting that JMTV might have complex transmission routes. Overall, current study, for the first time, reported that JMTV is prevalent in Jiangxi Province and provided additional information concerning JMTV distribution and evolution in China.

## 1 Introduction

Jingmen tick virus (JMTV) was initially discovered by Dr. YZ Zhang’s group in the ticks (Ixodidae) samples collected at Jingmen City, Hubei Province, China in 2014 (1). The genome of JMTV is segmented Positive-sense single-stranded RNA (+ssRNA viruses), consisting of four distinct segments. Segments 1 and 3 of JMTV encode non-structural proteins NSP1 and NSP2, which shared resemblance with the NS5 protein and NS2b/NS3 complex of flaviviruses (1, 2). Surprisingly, segments 2 and 4, which might be viral structural proteins, appear to be unique and no homologous viral or animal sequence was identified in GenBank, suggesting the potential derivation from novel, yet unidentified sources (1). The distinctive genomic characteristics exhibited by JMTV are conducive to comprehend its possible intricate evolutionary history and obtain insights on the potential advantages of segmented viruses and their ancestors (3).

After the discovery of JMTV, more segmented flavi-like viruses, which all share high homology of NSP1 and NSP2 with JMTV (4), were discovered by viral-metagenomics sequencing and clustered within one unclassified group, naming Jingmenvirus group such as ALSV, Takachi virus, Xinjiang tick-borne virus, and Newport tick virus (5–9). Currently, the International Committee on Taxonomy of Viruses (ICTV) classifies the Jingmenvirus group within the Flaviviridae family.

Multiple evidences demonstrated that JMTV is a true arbovirus. JMTV has been detected in the salivary glands of male ticks in both natural and experimental infections (2, 10). Additionally, an analysis of JMTV codon usage preferences suggests its circulation between arthropod vectors and vertebrate hosts (9). Moreover, JMTV has been detected not only in adult ticks, but also in nymphs and unfed larvae, indicating potential vertical transmission (7). JMTV has been detected in various hosts, including ticks, buffalos, bats, and humans. Interestingly, the JMTV sequences collected from various host species were highly homologs, implying that JMTV might highly adapted too different hosts (1, 10, 11). However, the host spectrum and the cross-species transmission capability of JMTV have not been fully characterized.

Various clinical cases indicated a potential public health threat of JMTV. Na Jia et al. conducted high-throughput sequencing on 16 skin biopsy specimens of tick bite sites, resulting in the identification of four JMTV genome sequences (10). In four JMTV positive cases, which presented the mild symptoms including fever, headache, and myalgia, histopathology analysis revealed substantial coagulation necrosis and inflammatory infiltration. Severe cases required hospitalization due to high fever (10). In addition, Petra Emmerich et al. reported the presence of JMTV genome in the sera samples of three CCHF patients in Kosovo (12). Overall, the published cases manifested that JMTV is capable of infect various animals and humans. Further investigation of JMTV host ranges and pathogenesis is necessary. In addition to JMTV, Alongshan virus (ALSV), another member of Jingmenvirus group, was also reported in the blood of a human patient bitten by a tick in Inner Mongolia, China, showing substantial threats to public health (5).

In China, JMTV has been detected in various tick species, such as *Rhipicephalus microplus* (the positive rate is 53–63%) and *Haemaphysalis longicornis* (25.7–46.1%), in nine provinces across China, including Hubei, Fujian, and Yunnan et al. (1, 13, 14). Additionally, Jing-Jing Guo et al. have detected JMTV in buffalos and bats with positive rate 9.6 and 11.8%, respectively (11). Zhu-Mei Yu et al. reported that the positive rate of JMTV in livers of rodents in Xinjiang was 25.6% (15). However, the prevalence and distribution of JMTV in other 25 provinces and regions of China are still blank. Further, JMTV has been widely detected in ticks from the Americas (Brazil, 2016, and Trinidad and Tobago, 2017), Asia (Japan, 2020, Laos and Türkiye, 2019) Europe (France, Romania, 2019, and Serbia, 2020), and Africa (Kenya, 2019) (2, 16–18). Additionally, JMTV has been identified in buffaloes of Brazil and in red colobus monkeys of Uganda (2, 19). These studies demonstrated that JMTV has wide distribution, and broad host spectrum. The Pairwise distance analysis of these JMTV strains reported by Zhen Wu et al. showed that the nucleotide homologies of segment 1–4 of JMTV strains from different regions ranged from 69.4 to 100%, 57.4 to 100%, 68 to 99.9%, and 59.5 to 100%, respectively (18). These studies indicate that JMTV is conservative in local ecosystem however significant genetic diversities were detected in different countries and continents. Further epidemiological investigation of JMTV is necessary to profile their epidemiological status, transmission routes and genetic diversity, which might lay the groundwork for implementing appropriate monitoring, prevention and control measures of JMTV (20).

Jiangxi Province is located in the southeast of China. Due to abundant rainfall, lush vegetation, and diverse wildlife species, it provides a highly suitable habitat for wild boars and ticks in most areas of Jiangxi Province (21). However, investigation and research on tick-borne viruses in Jiangxi Province have never been reported. In addition, wild boars are important vectors for the interaction between humans and the wild ecological environment in Jiangxi Province. In this study, qRT-PCR was employed to investigate the prevalence of JMTV in ticks collected from wild boars in Jiangxi, and the JMTV genome sequences were obtained by Viral-metagenome. These data provided additional information of JMTV distribution and genetic background in Jiangxi Province, and highlighted the potential zoonotic threats of JMTV.

## 2 Method

### 2.1 Tick collection and identification

From 2020 to 2022, a total of 66 ticks were collected from the body surface of wild boars legally hunted in four cities of Jiangxi Province. The ticks were placed individually in separate collection tubes and brought back to the laboratory. They were photographed under the stereomicroscope for morphological identification, with reference to “Systematic taxonomy of ticks” (22). Each tick sample was washed with 75% ethanol to remove surface contaminants and then placed in a glass homogenizer for grinding. After adding 600 μL of Phosphate Buffered Saline (PBS), the mixture was centrifuged to collect the supernatant. The tick genomic DNA was extracted using a DNA extraction kit (TIANGEN, Tianjin, China), and the gene fragments of the 18S and internal transcribed space (ITS) of ribosomal DNA were amplified through PCR for further classification and identification of the ticks (23).

### 2.2 Determination of viral genome sequence and positive rate

The total tick samples were retrieved from the −80°C freezer, ground in liquid nitrogen, and added to the RNA isolation reagent (Vazyme, Nanjing, China) for total RNA extraction following the instructions provided. Primers and probe were designed based on the study by Yuli Zhang et al. to amplify the RNA fragment of JMTV segment 1 (14). The extracted total RNA was then used to detect the infection of JMTV in the samples by qRT-PCR. In addition, in the subsequent processes of JMTV genome assembly of viral metagenomic sequencing, segment 4 was incomplete. The absent fragment was 345 bp. PCR amplification and sanger sequencing were employed to complete the lacked region of segment 4. The primers (seg4F: tacgtagctcacagcgtgc and Seg4R: ctcggagatggaacgagat) of the lacking fragment were designed based on the reference sequences in GenBank. After amplification, the PCR products were subjected to sanger sequencing to complete the sequence of segment 4.

### 2.3 Virome analyses using next-generation sequence

The JMTV positive sample (WY01) was selected and sent to the sequencing company (Novogene, Beijing, China) for virome analysis in order to obtain the complete genome sequence. The brief description of the operation steps is as follows: the ribosomal RNA forming the total RNA of the simple is depleted by commercial reagent (NEBNext Globin & rRNA Removal Kit). The purified RNA is randomly broken into short segments of 250–300 bp, the first strand of cDNA is synthesized by using the fragmented RNA as a template and random oligonucleotide as a primer, and then PCR amplification is carried out according to the general library building method of NEB, and finally the library on the computer is obtained. Different libraries were pooled according to the effective concentration and the demand of target offline data, and then Illumina sequencing (PE250) was carried out. To guarantee the accuracy and reliability of subsequent data analysis, the raw data underwent preprocessing to acquire high-quality, filtered clean reads. These data were then compared with the host genome to remove host reads. Transcripts were spliced and assembled using Trinity (v2.6.6) (24). Using pollution-free data to splice and assemble transcripts, in order to reduce false positives, the spliced results will be compared with virus database, Nr/Nt and CDD database to keep as many virus sequences as possible (25).

### 2.4 Phylogenetic analysis

The complete nucleotide sequences and encoded amino acid (aa) sequences of JMTV strains used in the phylogenetic analysis were obtained from GenBank. The Clustal W method was utilized for multiple sequences alignment and to describe the substitutions of aa sites among the strains (26). The phylogenetic tree was inferred using the maximum likelihood method in IQ-Tree 2.2.2.6 (27). Best-fit model according to Bayesian information criterion is GTR + F + I + G4 (28). Branches with bootstrap (1,000 replicates) values higher than 70 are considered reliable. The MegAlign tool in DNAStar Lasergene 7.1 is utilized to calculate the nucleotide and aa homology of segment 1 among different JMTV strains (29).

## 3 Results

### 3.1 Tick collection and JMTV epidemiological investigation in Jiangxi Province

**Figure 1:**
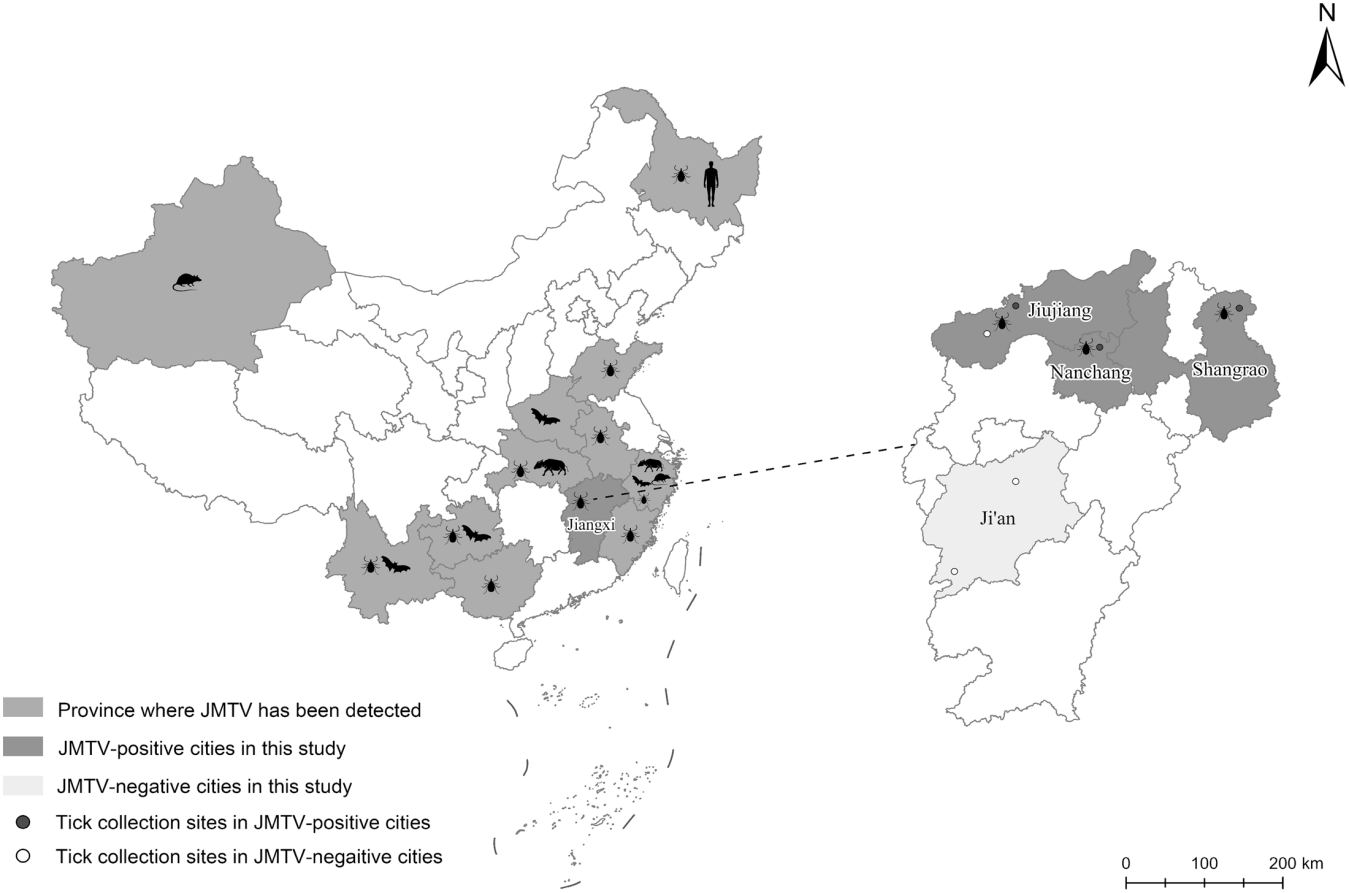
The distributions of JMTV reported in China and the locations of collecting ticks in current study. Totally 6 sampling locations in Jiangxi Province were showed as round dots in expanded map at right panel, of which red dots were JMTV positive location while white dots were JMTV negative location.

From 2020 to 2022, a total of 66 ticks were collected from 17 wild boars captured in four cities of Jiangxi Province (Figure 1). Through morphological and 18S and ITS rDNA determination, these ticks were found belonging to four tick species, including *Dermacentor silvarum*, *Haemaphysalis longicornis*, *Amblyomma testudinarium*, and *Dermacentor taiwanensis* (Table 1). As shown in Table 1, a total of 12 ticks from three cities were detected positive for JMTV by qRT-PCR. In this study, the overall JMTV-positive rate was 18.2% (12/66). The 12 JMTV positive ticks were collected from seven wild boars in current study. Specifically, ticks from Shangrao, Nanchang, and Jiujiang City exhibited positive rates of 27.3, 30, and 16.7%, respectively. However, no positive result was obtained from the samples collected in Ji’an City. The JMTV-positive rates of *D. silvarum* or *H. longicornis* were 33.3% (5/15) and 14.2% (7/48), respectively. These results indicated that *H. longicornis* is a dominant JMTV carrying tick species on the wild boars in Jiangxi Province. While the JMTV positive rate of *D. silvarum* was higher than *H. longicornis*, it should be noted that the samples were collected in north region of Jiangxi Province, and thus, it might not accurately represent the status in south region of Jiangxi Province.

**Table 1:** Detection of JMTV in ticks parasitized on wild boars in Jiangxi, China.

Capture position	Wild boar information	Tick species	Number of collections/positives	Positivity rate
Wuyuan County, Shangrao City	WY1/male	*D. silvarum* ^$^	5/3	27.3%
		*H. longicornis* ^&^	2/0	
	WY2/female	*D. silvarum*	4/2	
		*H. longicornis*	2/1	
	WY3/female	*H. longicornis*	3/0	
	WY4/male	*D. silvarum*	4/0	
		*H. longicornis*	2/0	
Xinjian district, Nanchang City	XJ1/female	*H. longicornis*	2/0	30%
	XJ2/female	*H. longicornis*	4/2	
	XJ3/male	*Amblyomma testudinarium*	1/0	
		*H. longicornis*	3/1	
Xiajiang County, Ji’an City	XAJ1/female	*H. longicornis*	5/0	/^*^
	XAJ2/male	*H. longicornis*	4/0	
	XAJ3/female	*H. longicornis*	1/0	
Suichuan County, Ji’an City	SC1/male	*H. longicornis*	2/0	/
	SC2/female	*H. longicornis*	4/0	
Wuning County, Jiujiang City	WN1/female	*H. longicornis*	5/2	25%
	WN2/male	*H. longicornis*	2/0	
	WN3/male	*D. silvarum*	2/0	
		*H. longicornis*	3/1	
Xiushui County, Jiujiang City	XS1/female	*D. taiwanensis*	2/0	/
	XS2/female	*H. longicornis*	4/0	
Overall positive rate	17 boars		66/12	18.2%

^*^The slash (/) indicates that no JMTV-positive samples were detected in the county. ^$^*D. silvarum* is short for *Dermacentor silvarum*; ^&^*H. longicornis* is short for *Haemaphysalis longicornis*.

### 3.2 Phylogenetic relationship between Jiangxi strain and other known JMTV strains

The JMTV positive sample WY01 collected in Wuyuan County were subjected to metagenomic sequencing. The assembly results were annotated, and four JMTV genome segments were recognized. The C terminal region of segment 4 were absent and was further determined by sanger sequencing of RT-PCR product. Finally, the complete coding sequence of four segments of WY01 strain (accession number: OR652603-OR652606) was obtained.

**Figure 2:**
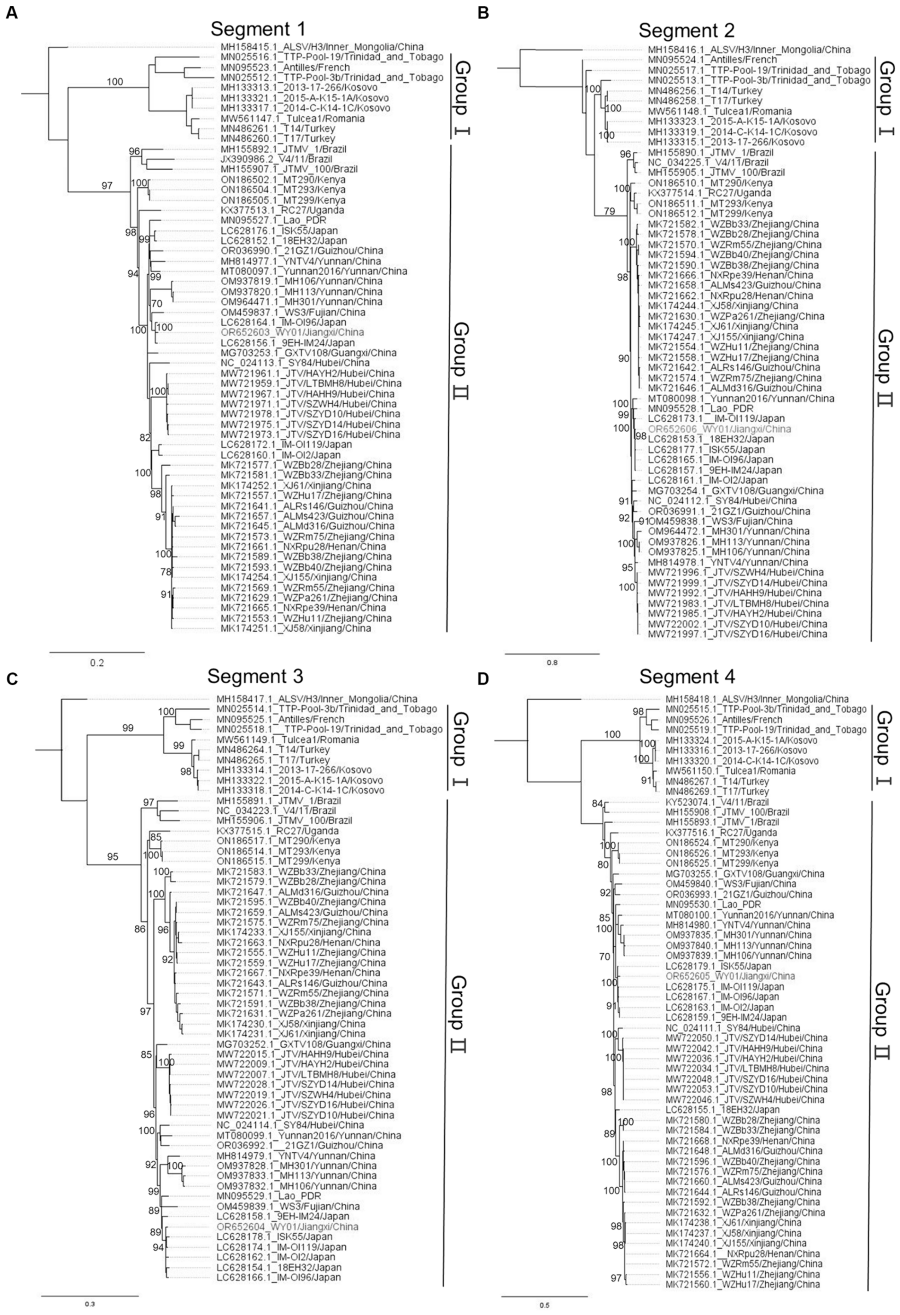
Phylogenetic tree of JMTV nucleotide sequence, panels **(A–D)** represent segments 1 to 4, respectively. The WY01 strain is marked in red font. The GenBank accession number, strain, and location of each sequence were noted. The phylogenetic tree was constructed using the maximum likelihood method implanted in IQ-tree 2.2.2.6. The percentages of replicate trees in which the associated taxa clustered together in the bootstrap test (1,000 replicates) were shown next to the branches. The trees were drawn to scale, with branch lengths in the same units as those of the evolutionary distances used to infer the phylogenetic trees.

In order to profile the genetic characteristics of WY01, multiple sequences alignment and phylogenetic analysis were carried out by pooling the WY01 sequence with a total of 58 reference sequences from GenBank. Alongshan virus, which also belongs to segmented flaviviruses and genetically close to JMTV, was set as the root in the phylogenetic tree. The phylogenetic trees of the four segments all showed two distinct lineages, Group I and Group II (Figure 2). Group I includes strains from Europe, Central America, and West Asia, while Group II consists of strains from Africa, South America, East Asia and Southeast Asia (Figure 2). Within Group II, Asia strains, Brazil strains, and Africa strains were divided in separated branches (Figure 2).

Surprisingly, the WY01 identified in Jiangxi Province was positioned closer to Japan strains such as IM-OI96 and 9EH-IM24 rather than other China strains. In comparison to the strains from China, segment 1 and 3 of WY01 show a closer evolutionary relationship with strain WS3 from Fujian Province, a neighboring province in the southeast of Jiangxi (Figures 2A,C). On the other hand, segment 2 and 4 exhibit a closer evolutionary relationship with strains from Yunnan Province, a southern border province far away from Jiangxi Province (Figures 2B,D).

NSP1 was the most conservative nonstructural viral protein in flavivirus, hence it was frequently used for phylogenetic analysis in flaviviruses. To further understand the genomic characteristics and genetic diversity of WY01 strains, calculations were performed on the nucleotide and aa homology of segment 1 (Table 2). The results showed, that compared with other JMTV sequences, the nucleotide identity between WY01 segment 1 and reference sequences ranged from 79.8 to 98.9%, while the aa homology ranged from 91.5 to 99.8%. According to the phylogenetic tree of segment 1 described in Figure 2A, the NSP1 protein of WY01 has 99.8% aa identity with the Japanese strain 9EH-IM24, which shows the closest evolutionary relationship with WY01. Furthermore, the nucleotide identity of segment 1 between WY01 and the strains from East Asia is 89.9–98.9%, whereas the identity between WY01 and the strains in Group I is 79.8–80.7% (Table 2). By comparing the aa sequences encoded by segment 1 of WY01 with 32 reference strains, all the mutation sites were displayed in Supplementary Figure 1. As shown in Supplementary Figure 1, Group I and Group II can be distinguished by 18 substitutions. NSP1 was predicted to contain two functional domains: the N terminal has the capping-2OMTase-viral domain which is responsible for catalyzing mRNA capping at the N terminal, while the C terminal has RNA dependent RNA polymerase (RdRp) domain which is essential for viral genome synthesis. In capping-2OMTase-viral domain, six distinguishing mutations (E133Q, R/G136K, E137D, R/H177Y, A204S, A216P) were found, meanwhile five distinguishing (L413Q, T837R, K870R, S/N/G882N, D889G) mutations were found in RdRp domain (Supplementary Figure 1). The substitutions of WY01 at the second aa residue (D/E2N) is a unique mutation, which is different from all reference strains.

**Table 2:** Homology of nucleotide and amino acid of JMTV segment 1.

Amino acid sequence identity^a^ (%)																													
	1	2	3	4	5	6	7	8	9	10	11	12	13	14	15	16	17	18	19	20	21	22	23	24	25	26	27	28	29
OR652603_WY01/Jiangxi/China		99.8	97.8	98.1	98.4	98.8	99.1	98.8	98.5	98.7	98.4	98.4	98.4	97.7	97.8	97.8	97.7	98.2	97.6	97.8	97.8	97.3	96.1	92.3	92.5	92.8	92.1	91.5	78.8
LC628156.1_9EH-IM24/Japan	98.9		98.0	98.4	98.6	99.0	99.3	99.0	98.7	98.9	98.6	98.6	98.6	97.9	98.0	98.0	97.9	98.5	97.8	98.0	98.0	97.5	96.3	92.2	92.3	92.9	92.3	91.6	79.0
OM459837.1_WS3/Fujian/China	96.2	96.5		94.2	90.6	97.7	95.5	97.7	96.9	97.2	96.9	97.2	96.9	96.5	96.6	96.6	96.5	97.0	96.4	96.6	96.6	96.2	94.6	90.8	90.9	91.5	90.7	90.2	77.7
OR036990.1_21GZ1/Guizhou/China	94.1	94.3	97.0		97.8	98.0	94.5	98.0	97.5	98.1	97.5	97.7	97.5	97.6	97.5	97.7	97.6	97.6	97.5	97.7	97.5	96.7	95.5	91.4	91.5	91.9	91.6	90.7	79.0
OM937821.1_MH203/Yunnan/China	91.2	91.4	97.0	92.0		98.2	91.6	98.1	97.9	97.7	97.7	97.7	98.0	97.6	97.7	97.7	97.6	97.8	97.5	97.7	97.7	96.7	96.3	92.3	92.1	92.7	92.3	91.7	79.3
MH814977.1_YNTV4/Yunnan/China	94.8	94.8	94.7	93.2	90.6		95.3	95.0	94.3	98.1	92.6	92.8	98.1	93.1	93.2	93.2	93.3	93.0	93.3	89.6	89.6	97.2	96.0	92.5	92.6	93.1	92.3	91.8	79.5
LC628176.1_ISK55/Japan	96.5	96.6	97.6	97.9	98.4	98.8		98.6	98.5	98.5	98.1	98.1	98.4	97.5	97.6	97.6	97.5	98.0	97.4	97.6	97.6	97.3	96.1	92.0	92.1	92.7	92.1	91.4	79.1
MT080097.1_Yunnan2016/Yunnan/China	95.6	95.8	95.0	93.9	91.0	98.9	96.2		98.1	98.1	93.3	93.3	98.1	98.1	98.2	98.2	98.1	98.5	98.0	98.2	98.2	97.2	96.2	92.0	92.1	92.7	92.1	91.4	79.5
MN095527.1_Am.testudinarium/Lao PDR	95.5	95.7	94.6	93.7	91.0	98.2	96.2	95.7		97.8	93.6	93.5	97.9	97.6	97.7	97.7	97.6	97.9	97.5	97.7	97.7	97.0	96.1	92.6	92.7	93.0	92.7	91.9	79.2
MG703253.1_GXTV108/Guangxi/China	95.2	95.5	94.6	93.5	90.3	93.8	95.7	94.9	94.8		93.7	93.7	97.9	93.5	93.7	93.6	93.8	93.6	93.7	89.9	89.9	96.8	95.6	91.4	91.7	92.2	91.9	90.9	78.9
MW721978.1_JTV/SZYD10/Hubei/China	94.2	94.3	93.6	91.8	88.5	98.1	94.1	98.1	98.1	97.9		99.8	98.1	97.8	97.9	97.9	97.8	98.1	97.7	98.0	98.0	96.9	95.8	92.0	92.3	93.1	92.2	91.6	79.4
MW721967.1_JTV/HAHH9/Hubei/China	94.2	94.3	93.5	91.8	88.5	98.4	94.1	98.4	98.1	97.9	99.3		98.1	98.0	98.1	98.1	98.0	98.4	97.9	98.2	98.2	97.2	95.8	91.8	92.1	92.9	92.1	91.4	79.5
LC628160.1_IM-OI2/Japan	94.6	94.8	93.9	92.4	89.9	93.3	95.1	94.3	94.4	94.8	94.1	94.0		94.1	94.2	94.3	94.4	94.1	94.3	90.5	90.6	97.3	96.1	92.1	92.6	92.8	92.7	91.8	79.5
MK721661.1_NXRpu28/Henan/China	93.6	93.8	92.9	92.1	89.2	97.7	93.6	93.5	93.6	97.6	92.9	92.9	97.7		99.7	99.9	99.8	98.8	99.9	99.9	99.9	96.7	95.6	92.0	92.3	92.8	92.6	91.6	79.4
MK721657.1_ALMs423/Guizhou/China	93.7	93.9	93.0	91.7	89.2	97.8	93.8	93.6	93.8	97.5	93.1	93.1	97.8	98.4		99.6	99.5	99.1	99.6	99.6	99.8	96.8	95.7	92.1	92.5	92.9	92.7	91.7	79.3
MK721629.1_WZPa261/Zhejiang/China	93.6	93.9	93.1	92.1	89.1	97.8	93.8	93.6	93.8	97.7	93.2	93.2	97.8	99.4	98.4		99.9	98.9	99.8	99.9	99.8	96.8	95.7	92.1	92.5	92.9	92.7	91.7	79.5
MK721593.1_WZBb40/Zhejiang/China	93.8	93.8	93.8	93.8	93.8	97.7	93.8	93.8	93.8	97.6	93.8	93.8	97.7	93.8	93.8	93.8		98.8	99.7	99.9	99.7	96.7	95.6	92.0	92.3	92.8	92.6	91.6	79.4
MK721577.1_WZBb28/Zhejiang/China	93.6	93.5	92.7	91.5	89.4	98.2	94.0	93.4	93.4	97.8	93.0	93.1	98.0	95.5	96.8	95.5	95.6		98.9	98.9	98.9	97.5	96.2	92.2	92.6	92.8	92.6	91.8	79.2
MK721573.1_WZRm75/Zhejiang/China	93.8	94.0	93.1	92.0	89.1	97.6	93.8	93.6	93.8	97.5	93.1	93.1	97.6	99.7	98.5	99.5	99.7	95.6		99.8	99.8	96.6	95.5	91.9	92.2	92.7	92.5	91.5	79.4
MK174254.1_XJ155/Xinjiang/China	89.9	90.2	89.4	90.8	91.4	97.8	90.0	89.9	90.1	97.7	89.5	89.5	97.8	95.9	94.6	95.6	95.6	91.9	95.8		99.8	96.8	95.7	92.1	92.4	93.0	92.7	91.7	79.6
MK174252.1_XJ61/Xinjiang/China	90.1	90.3	89.5	90.8	91.5	97.8	90.1	89.9	90.2	97.5	89.6	89.6	97.8	95.7	94.6	95.4	95.4	91.9	95.6	99.7		96.8	95.7	92.1	92.4	93.0	92.7	91.7	79.5
KX377513.1_RC27/Uganda	92.4	92.7	91.7	90.5	88.3	91.4	92.7	92.1	91.9	92.4	91.2	91.2	92.1	90.7	90.7	91.0	91.1	91.0	90.9	87.2	87.2		95.7	92.0	92.1	92.7	92.0	91.6	79.0
JX390986.2_MGTV/V4/11/Brazil	89.1	89.3	88.4	87.1	85.5	88.3	89.1	89.2	88.6	89.1	87.6	87.5	88.8	87.4	87.7	87.6	87.7	87.9	87.6	84.0	84.1	89.0		91.8	91.9	92.8	92.6	91.1	78.9
MH133321.1_2015-A-K15-1A/Kosovo	80.7	80.7	80.0	78.5	77.3	80.1	80.2	79.6	80.5	80.0	80.0	80.0	80.2	79.9	80.0	79.9	79.9	80.3	79.9	76.9	76.8	80.2	80.2		98.4	94.9	93.9	98.2	78.2
MW561147.1_Tulcea1/2019/Romania	80.3	80.3	79.7	78.3	77.2	80.1	80.3	79.6	80.1	79.8	79.5	79.4	79.8	79.7	79.9	79.7	79.7	80.0	79.7	76.8	76.7	80.0	80.3	95.8		94.6	94.3	98.4	78.3
MN025516.1_TTP-19/Trinidad and Tobago	80.0	80.5	79.9	79.0	77.6	80.5	80.3	80.2	79.9	80.5	80.1	79.9	80.5	80.0	80.1	80.0	80.0	79.8	80.0	76.9	76.8	80.6	80.3	86.2	85.9		95.3	94.0	78.9
MN095523.1_2014/2015/Antilles/French	79.8	79.9	79.2	78.6	77.3	80.1	79.7	79.3	79.8	79.3	79.6	79.4	79.7	79.8	79.9	79.8	79.9	79.6	79.9	76.9	76.8	80.2	79.8	86.3	86.3	87.0		93.5	79.2
MN486261.1_T14/Türkiye	79.8	80.1	79.2	78.0	76.8	79.4	79.8	79.0	79.7	79.4	79.1	79.1	79.4	79.2	79.3	79.1	79.2	79.4	79.1	76.1	76.1	79.4	79.7	96.0	96.3	85.5	85.9		77.9
MH158415.1_Alongshanvirus/H3/China	71.0	71.1	70.5	70.3	69.4	71.1	70.9	71.1	71.4	70.9	70.9	70.7	70.9	70.6	70.5	70.7	70.7	70.7	70.7	69.9	69.8	70.8	71.2	71.1	71.1	71.4	71.0	70.8	
Nucleotide sequence identity^a^ (%)																													

^a^Bottom diagonal is nucleotide identity and top diagonal is amino acid identity.

## 4 Discussion

Since JMTV first reported in 2014, JMTV has been detected in various hosts around the globe (1, 2, 12, 16, 19). The reports of JMTV in multiple provinces within China suggested its wide distribution throughout different regions of the country (11, 14, 15, 30). Jiangxi and Fujian Province have the highest forest coverage and possesses relatively high biodiversity in China. Therefore, both provinces also face the potential public health risks of tick-borne virus storage and transmission (31, 32). However, the epidemiological status of JMTV in Jiangxi Province is still unknown. In this investigation, the positive rate of JMTV was around 18.2%. This result indicated that JMTV is prevalent in ticks collected from wild boars in Jiangxi Province. The JMTV positive rate in current study is substantially lower than the positive rates reported in both east neighboring provinces Fujian and Zhejiang Province (11, 14). It is worth noticing that JMTV investigations of most provinces reported in China were positive except the investigation in Tibet, highlighting the wide spread of JMTV in China (33).

While the experimental proof is currently lacking, there are common speculations in existing reports that suggested vertebrate infection with JMTV may occur through horizontal transmission via tick vectors feeding on hosts (4). Additionally, considering the diverse lifecycles of different tick species and hosts, tick-mediated transmission of JMTV could potentially be highly complex (4). It is worth noting that, in this study, we detected JMTV in the host *D. silvarum*. Lian-Feng Li et al. reported infected *D. silvarum* with JMTV by the immersion technique to detect the transmission of JMTV, and it was found that *D. silvarum* may not highly susceptive to JMTV infection (34). Our results have provided new evidence for the capability of *D. silvarum* carrying JMTV. Additionally, *D. silvarum* are mainly distributed in the north of China (35). The collection of these ticks in Jiangxi may be due to the changes in suitable habitat. Studies have shown that global climate change, including changes in temperature and precipitation, may lead to an eastward shift in the suitable habitat of the *D. silvarum* (36). The distribution of *Hyalomma* spp. has been reported to be related to the incidence of CCHFV infection (37). Consequently, these findings highlighted the necessity of enhancing epidemiological investigation to profile the potential risks associated JMTV transmission.

To characterize the genetic background of JMTV Jiangxi Strains, WY01, discovered in Wuyuan County, was subjected to metagenomic sequencing. Interestingly, phylogenetic analysis revealed that WY01 exhibits a closer genetic relationship with the strain detected in Japan rather than the strains identified in neighboring provinces such as NXRpu28 and WZRm75 (Figure 2). The nucleotide homology between segment 1 of WY01 strain and the Japanese strains (94.6–98.9%) are higher than that between the strains from Henan and Zhejiang provinces (93.6–93.8%) (Table 2). This phenomenon might resulted from long distance JMTV with unknown routes. The natural geographical isolations between Japan and the coastal areas of China and their location on the migratory route of birds in the northern hemisphere also suggest the potential for tick-mediated transmission facilitated by migratory birds, which has been reported in the case of other tick-borne viruses (7, 38). We speculated that infected birds might be parasitized by ticks that could transmit the pathogen across the sea. Also, there are other potential transmission routes, such as the international animal trade and transportation vehicles. Further investigations are required to elucidate which transmission routes contributed to JMTV long distance transmission. Meanwhile, the strains from the same province in China exhibit a relatively higher genetic homology, implying a localized geographic clustering on a small scale. However, the evolutionary relationship between strains in different provinces is not completely associated to the geographical distances between provinces. Similarly, Zhu-Mei Yu et al. found strains in Xinjiang Province of China that shared closer genetic relationships with sequences from Hubei and Zhejiang Provinces, which were thousands of kilometers away from Xinjiang Province (15). Further analysis suggested that strain WS3 might has segments reassortment (data not shown). Overall, the genetic clustering was significantly associated with geographical clustering at continent level, however the discrepancy was found in smaller scale such as the discrepancy found in the China and Japan strains.

The NS5 is the most conserved protein encoded by flaviviruses. In JMTV, the NS5-like protein encoded by segment 1 has been widely used for genetic and evolutionary analysis (39). By comparing the mutation sites of the NS5-like protein among JMTV strains, the distinguishing mutations were observed between the two genotypes in the NSP1 coding region. Yet, the impacts of these variations remain unclear. Overall, current study, for the first time, disclosed that JMTV is prevalent in ticks collected from wild boars in Jiangxi Province implying potential threats to public health. The investigation scale was limited in this study which may lead to bias in determining genetic variability of the virus. In the future, more comprehensive epidemiological investigation is necessary to enhance our understanding of its distribution, host spectrum and evolution.

## Data availability statement

The datasets presented in this study can be found in online repositories. The names of the repository/repositories and accession number(s) can be found at: https://www.ncbi.nlm.nih.gov/genbank/, OR652603; https://www.ncbi.nlm.nih.gov/genbank/, OR652604; https://www.ncbi.nlm.nih.gov/genbank/, OR652605; https://www.ncbi.nlm.nih.gov/genbank/, OR652606.

## Ethics statement

The animal studies were approved by the Ethics Committee of Jiangxi Agricultural University. The studies were conducted in accordance with the local legislation and institutional requirements. Written informed consent was obtained from the owners for the participation of their animals in this study.

## Author contributions

ZL: Conceptualization, Data curation, Formal analysis, Investigation, Validation, Visualization, Writing – original draft, Writing – review & editing. RH: Conceptualization, Data curation, Formal analysis, Methodology, Project administration, Software, Supervision, Validation, Writing – review & editing. HC: Conceptualization, Funding acquisition, Project administration, Resources, Supervision, Validation, Writing – review & editing. PH: Project administration, Resources, Supervision, Writing – review & editing. HY: Project administration, Resources, Supervision, Writing – review & editing. PM: Investigation, Project administration, Resources, Writing – review & editing. ZX: Data curation, Investigation, Writing – review & editing. XD: Data curation, Writing – review & editing. FY: Data curation, Formal analysis, Writing – review & editing. LW: Investigation, Writing – review & editing. QQ: Methodology, Validation, Writing – review & editing. LY: Methodology, Validation, Writing – review & editing. TZ: Investigation, Resources, Writing – review & editing.
