# Supplementary material for: Identification and phylogenetic analysis of Jingmen tick virus in Jiangxi Province, China
Source: Front Vet Sci. 2024 May 2;11:1375852. doi: 10.3389/fvets.2024.1375852 (PMC11096534; doi:10.3389/fvets.2024.1375852)
Supplement: Supplementary file 1 [file Image_1.pdf]

Supplementary Material

1 Supplementary Figures

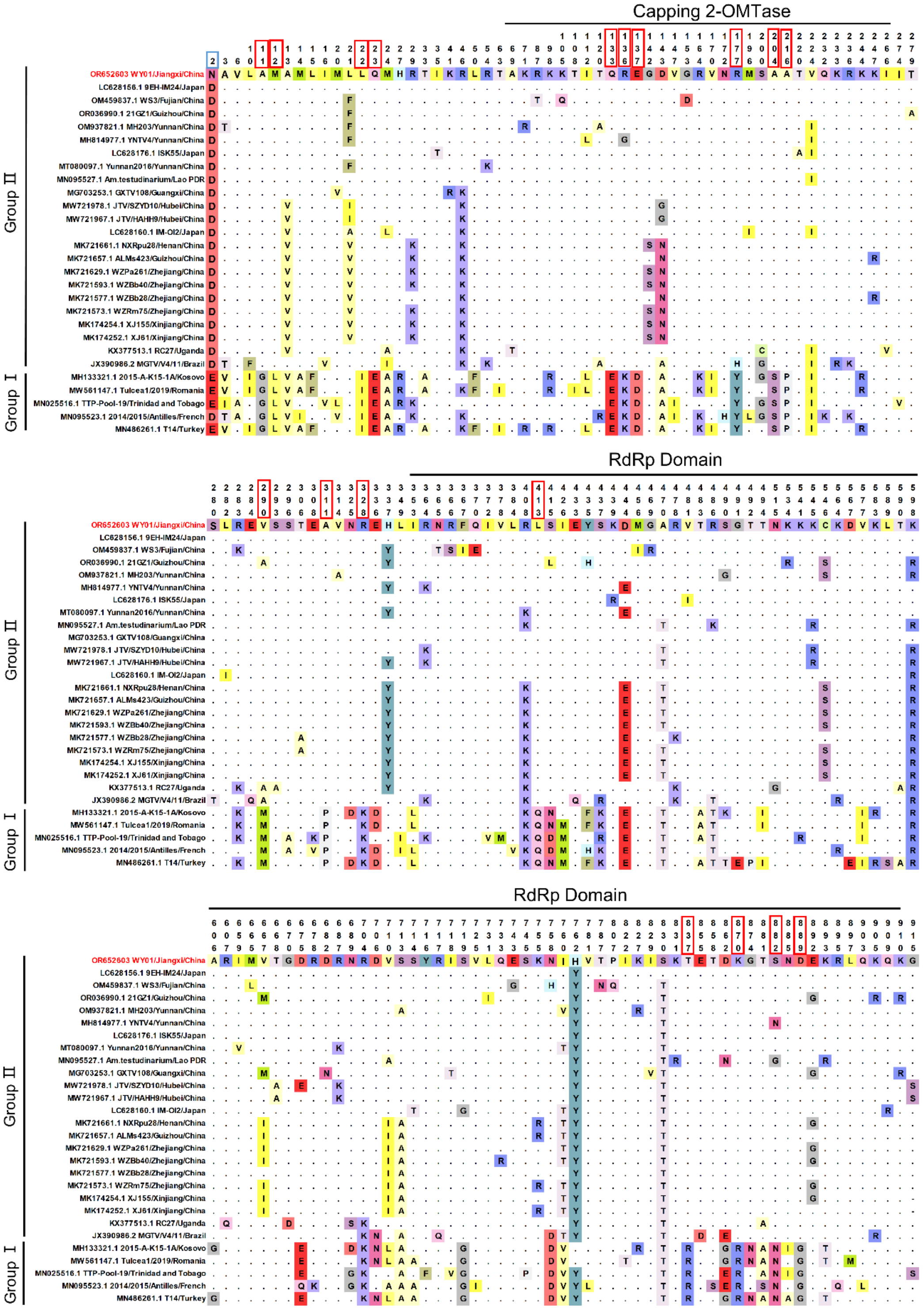

Supplementary Figure 1. Substitutions of amino acid sites in the NSP1 protein among JMTV strains. Multiple sequence alignment by Clustal W method. The WY01 strain obtained in this study is marked in red font. Eighteen amino acid substitutions that can distinguish the Group I and Group II are marked by red frames. The unique mutation of WY01 at site 2 is indicated by a blue frame.
